# Behavioral responses of *Rhodnius prolixus* to volatile organic compounds released *in vitro* by bacteria isolated from human facial skin

**DOI:** 10.1371/journal.pntd.0006423

**Published:** 2018-04-23

**Authors:** Marcela Tabares, Mario Ortiz, Mabel Gonzalez, Chiara Carazzone, Martha J. Vives Florez, Jorge Molina

**Affiliations:** 1 Centro de Investigaciones Microbiológicas (CIMIC), Universidad de los Andes, Bogotá, Colombia; 2 Centro de Investigaciones en Microbiología y Parasitología Tropical (CIMPAT), Universidad de los Andes, Bogotá, Colombia; 3 Laboratory of Advanced Analytical Techniques in Natural Products (LATNAP), Universidad de los Andes, Bogotá, Colombia; Tulane University School of Public Health and Tropical Medicine, UNITED STATES

## Abstract

**Background:**

Previous studies have demonstrated the role of volatile organic compounds (VOCs) produced by skin microbiota in the attraction of mosquitoes to humans. Recently, behavioral experiments confirmed the importance of VOCs released by skin microbiota in the attraction of *Rhodnius prolixus* (Hemiptera: Triatominae), a vector of Chagas disease.

**Methods/Findings:**

In this study, we screened for VOCs released *in vitro* by bacteria isolated from human facial skin that were able to elicit behavioral responses in *R*. *prolixus*. The VOCs released *in vitro* by eight bacterial species during two growth phases were tested with adult *Rhodnius prolixus* insects using a dual-choice “T”-shaped olfactometer. In addition, the VOCs released by the bacteria were analyzed with headspace solid-phase microextraction gas chromatography-mass spectrometry (HS-SPME-GC-MS). The VOCs produced by *Staphylococcus capitis* 11C, *Staphylococcus warneri* and *Staphylococcus epidermidis* 1 were attractive to *R*. *prolixus*, while the VOCs released by *Citrobacter koseri* 6P, *Brevibacterium epidermidis* and *Micrococcus luteus* 23 were non-attractive.

**Conclusions:**

The results shown here indicate that VOCs released by bacteria isolated from human facial skin have a potential for biotechnological uses as a strategy to prevent the vectorial transmission of Chagas disease mediated by *Rhodnius prolixus*.

## Author summary

Volatile organic compounds released by bacteria growing on human skin are mediating human-blood-sucking insects interactions. In this study we were interested in those volatile organic compounds produced *in vitro* by skin bacterial metabolism that are involved in the *Rhodnius prolixus* interaction with humans. *Rhodnius prolixus* is the main vector of Chagas disease in Northern South America. Using behavioral experiments, chemical analysis and microbiological techniques we determined the volatile organic compounds released *in vitro* by bacteria isolated from human faces and its effects on *Rhodnius prolixus*. We found that most of the *Staphylococcus* species tested here are mediating attraction, while non-*Staphylococcus* species are non-attractive. As a conclusion, our results showed that volatile organic compounds released by bacteria isolated from human faces have a potential for biotechnological uses as a strategy to control the vectorial transmission of Chagas disease mediated by *Rhodnius prolixus*.

## Introduction

Human skin is colonized by a wide variety of beneficial microorganisms that inhibit the growth of pathogens and promote the processing of proteins and free fatty acids on the skin [1, 2]. The genera most frequently reported to be present on human skin are *Pseudomonas*, *Janthinobacterium* (phylum Proteobacteria), *Corynebacterium*, *Kocuria*, *Microbacterium*, *Propionibacterium*, *Micrococcus* (phylum Actinobacteria), *Staphylococcus*, *Clostridium* (phylum Firmicutes), and some species of the phyla Bacteroidetes, Cyanobacteria and Acidobacteria [1]. Woesearchaeota, Thaumarchaeota and Aenigmarchaeota are some Archaea also reported on human skin [3].

In addition, the human skin microbiota play an important role in the generation of human odors [2, 4, 5]. Human odor profiles include more than 350 identified compounds [6, 7], and it has been shown that bacteria on human skin are involved in the release of approximately 150 volatile organic compounds (hereafter referred to as VOCs) [8]. Some of these VOCs released by bacteria are typically found in human odor [2].

It has also been shown that VOCs released by bacteria are playing a role in attracting blood-sucking insects [5, 9]. Differential attraction to different human body parts has been observed; for instance, *Anopheles atroparvus* are attracted to faces, and *Anopheles gambiae* are attracted to feet and ankles [10]. In addition, it has been shown that this differential attraction of *Anopheles* to humans is mediated by the VOCs produced by bacterial species and is dependent on the growth phase of the bacteria [10, 11]. The combination of VOCs produced during the stationary growth phase of *Corynebacterium minutissimum*, *Staphylococcus epidermidis*, *Brevibacterium epidermidis* and *Bacillus subtilis* was attractive to *An*. *gambiae* in behavioral experiments [11].

Triatomines (Hemiptera: Reduviidae) are known as kissing bugs because of their preference to bite human faces [12]; triatomines have been widely studied because some species are involved in the vectorial transmission of Chagas disease, which is caused by the parasite *Trypanosoma cruzi* [13]. Chagas disease is a public health problem in 17 countries of Latin America [13]. In Colombia, the prevalence of Chagas disease is estimated to be approximately 5% [14], and the main species of triatomines involved in the transmission of this disease are *Rhodnius prolixus*, *Triatoma dimidiata*, *Triatoma maculata* and *Triatoma venosa* [15].

The attraction of *R*. *prolixus* to vertebrates is due to factors such as CO_2_, heat, humidity and chemical compounds released during sweating [16]. However, some years ago, it was found that *R*. *prolixus* is strongly attracted to odor extracts from human faces and feet [17]. In addition, the differential attraction to different body parts was apparently related to the presence of VOCs produced by bacteria [17, 18].

Based on this preliminary evidence and keeping in mind the results obtained with *Anopheles*, we suggest that the attraction of *R*. *prolixus* to the human face could be a result of the VOCs produced by the bacterial microbiota present on facial skin. The aim of this study was to demonstrate the effect of VOCs produced *in vitro* by eight bacterial species (in exponential and stationary phases of growth) isolated from human skin on the attraction of *Rhodnius prolixus* using behavioral experiments. Finally, using gas chromatography coupled to mass spectrometry, we identified some of the VOCs produced *in vitro* by the bacterial strains tested behaviorally with *R*. *prolixus*.

## Methods

### Bacteria

Bacteria were isolated from the facial skin of ten volunteers living in Bogotá (5 men and 5 women between 20 and 35 years old) following the method described in [8]. Briefly, an acrylic plate (1.0 cm thick, diameter 2.9 cm) was placed on one of the cheeks of each volunteer. Then, 0.75 mL of full-strength buffer (75 mM sodium phosphate buffer [pH 7.9] + 0.1% (v/v) Triton X-100; Merck, The Netherlands) was added, and the surface of the skin was scrubbed for 1 min with a glass stick. The fluid (containing the skin bacteria) was collected, and the procedure was repeated two more times on the same area of the face. Pooled samples were diluted 5X in half-strength buffer (75 mM sodium phosphate buffer [pH 7.9] + 0.05% (v/v) Triton X-100; Merck, The Netherlands).

According with Colombian laws (Resolución 8430–1993), Ethics Committee from Universidad de los Andes approved the research project and categorized it as a non-risk project (Acta 159–2012). Oral consent to isolated bacteria from the human skin was obtained from volunteers after explaining the main objectives of the project and also that the process of isolation does not include neither reagents that are toxic or dangerous to health, nor invasive procedures. Finally the data were coded to ensure that they were analyzed anonymously.

Serial dilutions of the pooled samples from each volunteer were cultured on Columbia blood agar (Oxoid) and incubated at 37°C for 24 h [8]. Additionally, the samples were cultured on a new medium for culturing *Propionibacterium* [19] and incubated at 37°C for 7 days in anaerobic jars with AnaeroGen Gas Pack 150 (Oxoid).

Several isolated bacterial morphotypes were transferred successively until pure cultures were obtained. The isolates were identified by amplification of the 16S gene using universal primers 27F (AGAGTTTGATCCTGGCTCAG) and 1492R (GGTTACCTTGTTACGACTT), and the amplicons were sequenced by Macrogen, Korea. Sequences obtained were analyzed using BLASTn [20] and the Ribosomal Data Project (RDP) [21]; only sequences with a percent identity of 97% or higher were considered.

Six bacterial strains were selected for use in behavioral experiments with *Rhodnius prolixus* (S1 Table, S1 Fig): *Staphylococcus epidermidis* 1, *Staphylococcus caprae* 7P, *Staphylococcus capitis* 11C, *Citrobacter koseri* 6P, *Micrococcus luteus* 23 and *Dermacoccus nishinomiyaensis* 9C. The following criteria were used to choose the strains to be tested: To have at least one strain of all species isolated, to avoid testing two strains isolated from the same volunteer, to include strains with the highest identity (S1 Table), and finally, to use only those strains with the best *in vitro* growing. The bacterial strains *Staphylococcus warneri* and *Brevibacterium epidermidis* donated by Niels Verhulst were also included in the behavioral experiments.

### Growth curves

To identify the specific times at which each bacterium reaches the exponential or stationary phase, growth experiments were conducted as follows: For each bacterial strain, 12-h cultures in 4 mL of standard liquid medium (Infusion from heart muscle 2 g/L; peptone 13 g/L; yeast extract 5 g/L; sodium chloride 5 g/L; agar bacteriological 15 g/L, all reactives from Oxoid and distilled water 1,000 mL) [11] were incubated at 37°C, with shaking at 200 rpm. Then, 250 μL of each culture was transferred to a 125-mL Erlenmeyer flask with 25 mL of standard liquid medium. One-milliliter samples were extracted, and the absorbance of the samples at 620 nm was measured using a spectrophotometer (ThermoSpectronic BIOMATE 3); serial dilutions were plated on standard solid medium and incubated at 37°C for 24 h. The absorbance measurements were performed until each of the bacterial strains reached the stationary growth phase. Absorbance and log (CFU/mL) were plotted as a function of time (S2 Fig). The results of these growth curves allowed for the identification in each bacterial strain of the time at which half of the growth phase was reached. That time allowed us to carry out the behavioral experiments with 20 adults of *Rhodnius prolixus* on the olfactometer without to reach the next growing phase (see below).

### Insects

Male and female *Rhodnius prolixus* were obtained from a breeding colony maintained at Universidad de los Andes since 1979. Rearing conditions were as follows: 27 ± 2°C, 75 ± 10% relative humidity and an inverted circadian rhythm (6:00 h/18:00 h, dark/light; artificial illumination). The insects were fed monthly *in vivo* with hen's blood. Males and females with body weights of 0.0381 ± 0.002 g (approximately 15 days of starvation) were used to conduct the behavioral experiments [16].

### Olfactometer and behavioral experiments

**Fig 1:**
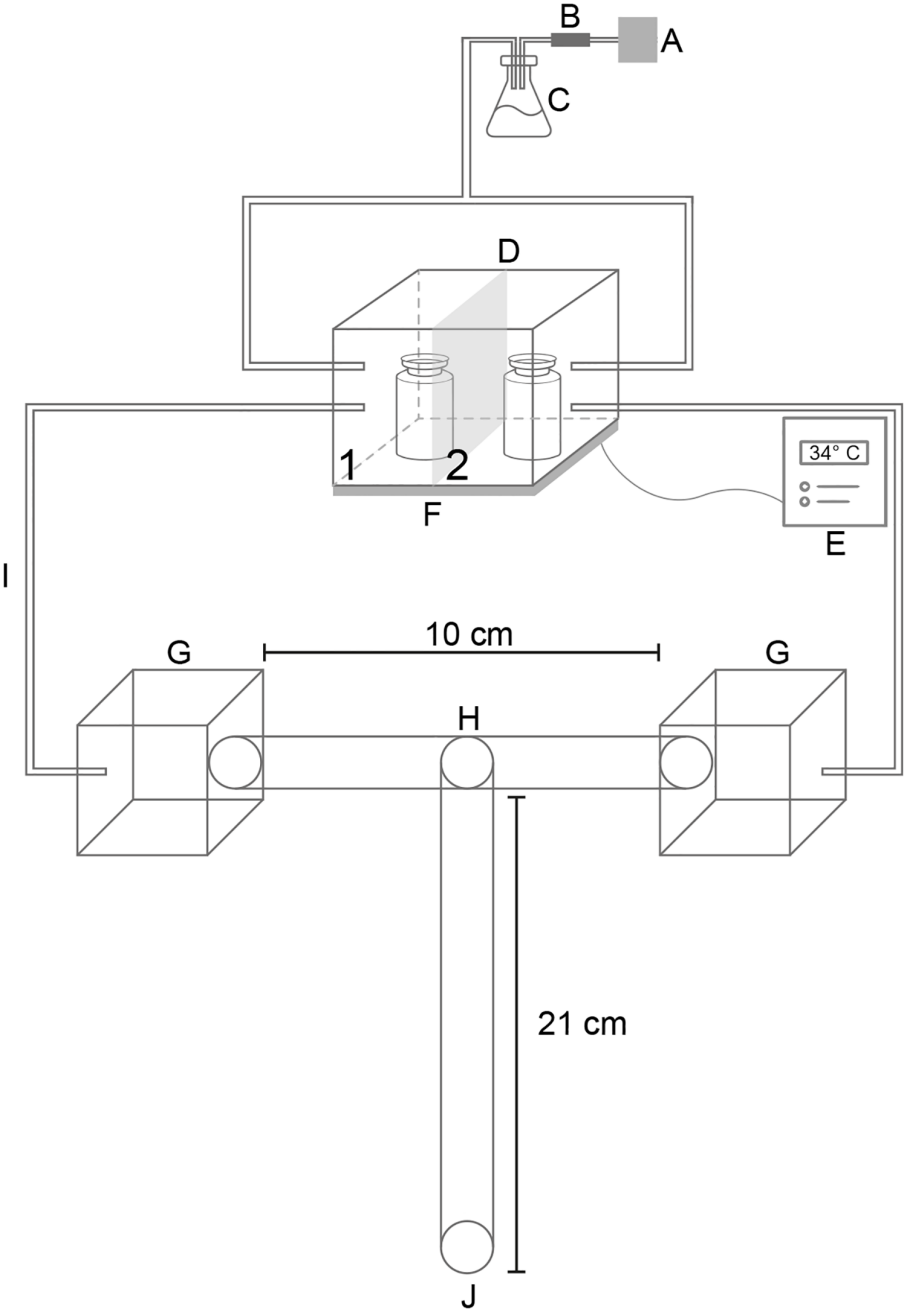
“T”-shaped olfactometer used to evaluate behavioral responses in *Rhodnius prolixus* to VOCs released *in vitro* by bacteria. A. Air pump. B. Charcoal filter. C. Erlenmeyer flask to humidify the air flow. D. Hermetic box with two sides (1: vial with standard liquid medium containing bacteria in exponential/stationary phase, 2: vial with standard liquid medium without bacteria). E. Thermostat. F. Hot plate. G. Hermetically sealed transparent packages. H. Insect decision point. I. Transparent hoses (airflow connected with the olfactometer). J. Insect starting point.

Behavioral experiments were conducted with a dual-choice “T”-shaped olfactometer modified from Ortiz & Molina (2010) [17] and Verhulst *et al*. (2009) [8]. The modified olfactometer consisted of an air pump with an airflow <10 cm/s measured with a thermo-anemometer (45118, EXTECH Instruments) between the humidifier and a charcoal filter (Fig 1). As shown before, the airflow is constant in both sides of the olfactometer and under this conditions, airflow can be excluded as a confounded variable affecting our results [17]. The humidified air was introduced through plastic tubes in an acrylic box (8.0 x 8.0 x 5.5 cm) that had two separated compartments. The base of the acrylic box was a galvanized metal plate with a regulated temperature of 34 ± 1°C, controlled with a pyrometer (0–400°C). Each of the two compartments of the acrylic box was connected with plastic tubes to one of the arms of the “T”-shaped olfactometer (Fig 1).

All behavioral experiments were carried out as follows: The insect was placed at the entrance of the “T”-shaped olfactometer, and a maximum of five minutes was given to the animal to choose one compartment of the olfactometer (Fig 1). Each insect was tested just once in a dimly lit room (light intensities averaged 0.3 μW/cm2 as measured with a radiometer/photometer ILT1400A; International Light Technologies Inc.) between 7:00 h and 10:00 h, which corresponds to the early scotophase of *R*. *prolixus* with an inverted circadian rhythm. Then, a trial starts by testing individually each insect, and finish when the insect is removed from the olfactometer. After testing 60–68 insects we finished an experiment. Each experiment consisted then of three replicates with 20 insects, and all individual components of the olfactometer were washed at least three times with water and gel soap and rinsed under a stream of water to eliminate the soap completely after complete each replicate. The olfactometer components were allowed to dry overnight [17].

For each of the different experiments, the glass vial in the compartment of the acrylic box contained 1 mL of standard liquid medium with a bacterial strain in either exponential or stationary phase of growth. The time necessary to test 20 insects (≤ 100 min), grant in our behavioral experiments that each bacterial strain continued its growth but without reaching the next growing phase (S2 Fig). For the next set of 20 insects, a new medium with bacteria of each species and growing phase was used. The glass vial in the second compartment of the acrylic box contained 1 mL of standard liquid medium without bacteria. After 20 trials, the compartments of the vials were switched to avoid any bias. We expected one of the following three results in our behavioral experiments with *R*. *prolixus*: Attraction (when insects choose non-randomly the side with the medium + bacteria), non-attraction (when insects choose non-randomly the side in the olfactometer with medium but without bacteria) and none (when insects choose randomly both sides of the olfactometer).

As a control, we tested the effect of VOCs emitted by the standard liquid medium used to culture the bacteria on the attraction of *R*. *prolixus*. For this control, one compartment of the acrylic box contained 1 mL of the standard liquid medium in a sterile glass vial, and the other compartment of the acrylic box contained a sterile glass vial. The glass vial with the standard liquid medium was switched between the two compartments after each replicate. The control standard liquid medium was tested with 63 insects, divided in three replicates. For each replicate, the control with standard liquid medium was changed to avoid any bacterial growth due to contamination from the environment. In control experiments also one of the following three results is expected: Attraction (when insects choose non-randomly the side with the medium), non-attraction (when insects choose non-randomly the side in the olfactometer without medium) and none (when insects choose randomly both sides of the olfactometer).

### Volatile organic compound (VOC) analysis by HS-SPME-GC-MS

VOCs from the standard liquid medium were analyzed for each of the eight bacterial strains in exponential and stationary phases of growth. Three replicate experiments were conducted for each bacterial species at each growth phase.

Each bacterial strain was cultured to initiate growth [11] and incubated at 37°C and 200 rpm for 18 h. Then, 250 μL of each culture was transferred to a 125-mL Erlenmeyer flask with 25 mL of standard liquid medium. One milliliter of the culture was extracted and its absorbance measured at 620 nm. When the bacteria reached the exponential (6 hours) or stationary (12 hours) phases of growth the VOCs produced were sampled with headspace SPME and subsequently analyzed by GC-MS.

The fiber (Supelco, Fiber SPME 85 μm CAR/PDMS, light blue) was exposed for 15 min to the headspace of the Erlenmeyer flask maintained at 37°C ± 2°C in order to capture the VOCs released by the bacteria. Then, the fiber was transferred to the GC-MS (Thermo, Trace 1300 gas chromatograph, ISQLT single quadrupole mass spectrometer). The GC-MS analysis was performed with a ZB-5 MSi GC column (HP-Zebron 30 m x 0.25 mm). The separation of the VOCs was achieved by using the following temperature profile: the temperature was initially maintained at 40°C for 1 min; then, the temperature was increased at a rate of 6°C/min until it reached 60°C, and then, the temperature was increased at a rate of 3°C/min until it reached 200°C; finally, the temperature was increased at a rate of 10°C/min until it reached 250°C for 12 min.

Splitless injection was performed using helium as the carrier gas at a constant flow rate of 1.0 mL/min. The mass spectrometer was operated in full-scan mode with a scan range of 35–300 m/z at a rate of 0.2 scans/s. The ion-source temperature was 250°C with an ionizing energy of 70 eV and a mass transfer line temperature of 280°C.

### Data analysis

The total number of insects that reached each one of the two compartments of the olfactometer was established at the end of each behavioral experiment and compared using binomial tests and a confidence interval of p < 0.05 [22]. All analyses were conducted using R software (version 2.11.1).

To analyze the GC-MS data, the profiles obtained in three replicates for VOCs from eight bacterial species were analyzed using the Qual Browser of Xcalibur 3.0 software (Thermo Scientific), and each peak with an S/N ratio over 3 was manually integrated. Only those peaks that were absent in the control analysis were integrated and considered to be volatile compounds released by bacteria. Compounds were annotated using NIST MS search 2.0 with the NIST 14 database.

The GC-MS data were processed to estimate the percentage of the area of each compound in every sample. Using the percentages of each replicate, a matrix was constructed, which reported annotated volatile compounds as columns/variables and estimated percentages in each chromatogram as rows/observations. As grouping variables, we included the eight bacterial species in both growth phases and the corresponding *R*. *prolixus* behavioral response (attraction/none/non-attraction). VOC profiles, including CO_2_, were analyzed using non-metric multi-dimensional scaling (NMDS) based on the Bray-Curtis distance matrix in order to reduce the complex data of the area percentages into a two-dimensional space. The closer the samples are in the NMDS, the more similar their VOC profiles. The reliability of the NMDS representation was estimated based on stress values, usually considered acceptable at a value less than 0.2. We used ANOSIM with 999 permutations in order to determine which grouping variable (species, growth phase, or both) better explained the differences in VOC profiles and to determine if this pattern could be related to the results obtained in the behavioral experiments with *R*. *prolixus*. We performed all statistical tests using R studio software (http://www.rstudio.org/) and the community ecology package vegan (http://CRAN.R-project.org/package=vegan). As the dataset contains many zeros, PCA analysis or NMDS using Euclidean distances were not appropriate because they can result in artifacts due to the lack of normal data distribution [23].

## Results

### Bacteria isolated from human facial skin

Fifty-five bacterial morphotypes were isolated from the facial skin of ten volunteers, but only 36 were identified by 16S sequencing (S1 Table). From these sequences, we identified seven species (S1 Fig). The only species isolated from the facial skin of all volunteers was *Staphylococcus epidermidis*. Interestingly, it was only possible to isolate four different species from two of the ten volunteers (S1 Fig).

From the species identified, we selected six species for growth analysis (S1 Fig). In addition, the donated strains *Staphylococcus warneri* and *Brevibacterium epidermidis* were included in the experiments (see Materials and methods). As a general result, we can highlight that all the species evaluated showed similar growth curves and reached the stationary phase at 12–14 hours (S2 Fig).

### Behavioral experiments with bacteria

**Fig 2:**
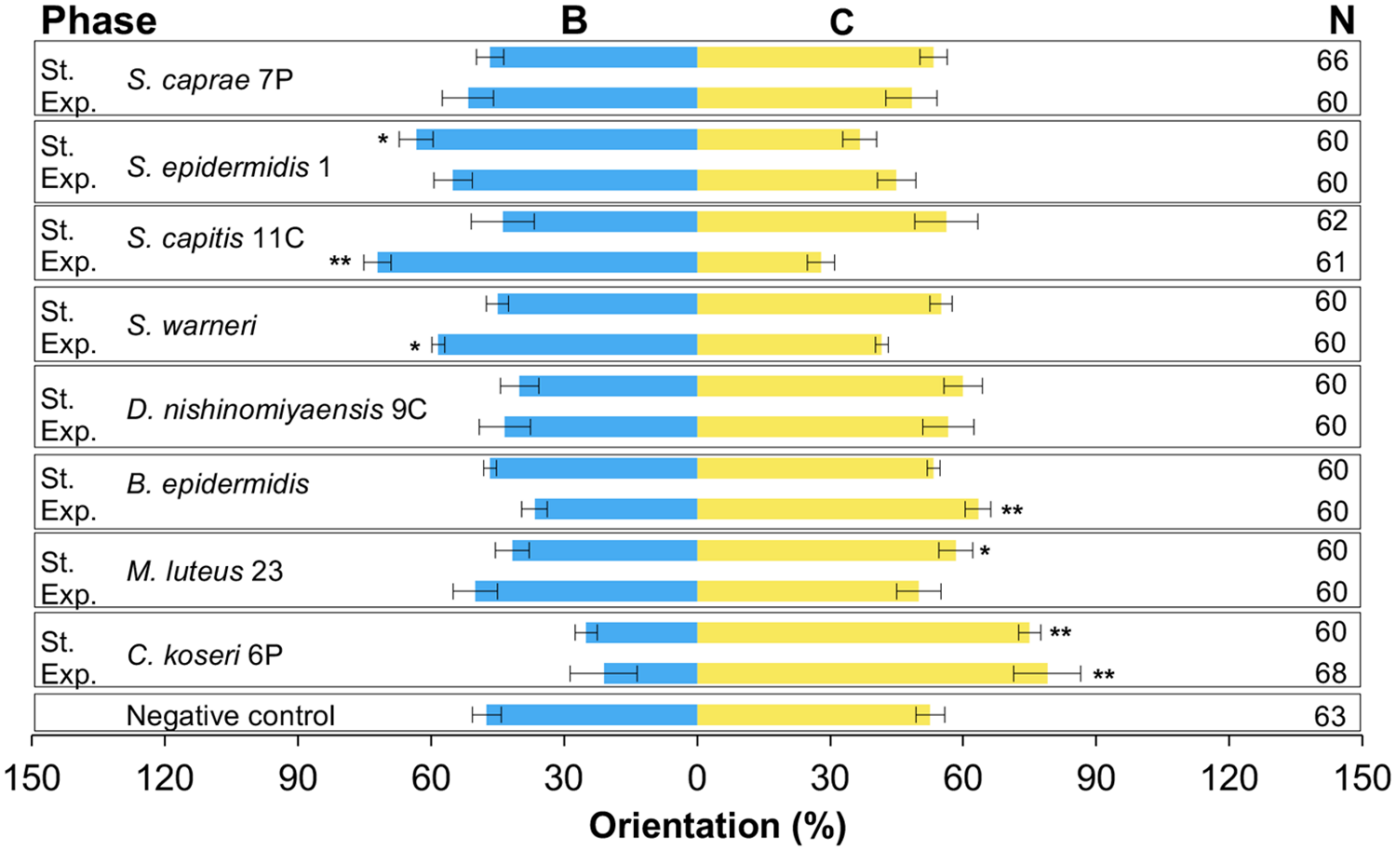
Behavioral responses of *R*. *prolixus* to VOCs produced *in vitro* by eight bacterial species. Bacterial species were tested in exponential growth phase (Exp) and in stationary growth phase (St). B = standard liquid medium with bacteria, and C = standard liquid medium without bacteria. N = Number of insects tested. *: P<0.05. **: P<0.01.

The control test showed that the *R*. *prolixus* were not attracted to the standard liquid medium (Fig 2, p = 0.093). In contrast, *R*. *prolixus* showed a clear attraction for the standard liquid medium containing three of the four *Staphylococcus* species evaluated (*S*. *capitis* 11C and *S*. *warneri* in the exponential phase and *S*. *epidermidis* 1 in the stationary phase) (Fig 2). On the other hand, *Citrobacter koseri* 6P, *Microccocus luteus* 23 and *Brevibacterium epidermidis* were non-attractive to *R*. *prolixus* in one or both growth phases (Fig 2).

Finally, in terms of behavioral response, *R*. *prolixus* was indifferent to the VOCs produced by the following bacteria: *S*. *caprae* 7P (exponential and stationary), *S*. *epidermidis* 1 (exponential), *S*. *capitis* 11C (stationary), *S*. *warneri* (stationary), *D*. *nishinomiyaensis* 9C (exponential and stationary), *B*. *epidermidis* (stationary) and *M*. *luteus* 23 (exponential) (Fig 2).

### Volatile organic compound (VOC) analysis by HS-SPME-GC-MS

Forty-six different VOCs were used to compare similarities between the percentage distributions among eight species of bacteria in two growth phases. We identified tentatively 34 VOCs by comparing experimental mass spectra with NIST14 Mass Spectral Library and analyzing the fragmentation patterns in EI. The annotation of functional groups was achieved for ten VOCs based on analysis of fragmentation patterns, but mass spectra similarity was insufficient to tentative identification. Annotation of each compound was carried out assigning retention times and spectral features based on mass spectra. Average area percentages for each species-phase combination are summarized in Table 1 organizing VOCs by retention times. *C*. *koseri* 6P is the species with the highest number of VOCs, followed by *M*. *luteus* 23, while *S*. *caprae* 7P has the lowest number. The only two VOCs shared by all eight species were CO_2_ and indole, which showed differences in the average percentage area between species in exponential and stationary phases.

**Table 1:** Tentative identification of VOCs released *in vitro* by the eight bacterial strains tested. VOCs are listed according to their retention times.

Tentative identification	Ret time (min)	Be_e_	Be_s_	Ck_e_	Ck_s_	Dn_e_	Dn_s_	Ml_e_	Ml_s_	Sc_e_	Sc_s_	Sk_e_	Sk_s_	Se_e_	Se_s_	Sw_e_	Sw_s_
**Carbon dioxide**_a_	2,43	+++	++	+	+	+++	+++	+++	++	+++	+++	+++	+++	++	++	+++	+++
**Methanethiol**_a_	3,15		+	+	+									+			
*Acetone_a_	3,82					+		+	++								
*Hexane_a_	5,76			+				+		+							
**Trichloromethane**_a_	6,30			+				+		+							
**1-Butanol_a_	7,44	+		+													++
**Methyl-thioacetate**_a_	8,81		+	+	+									+			
*Disulfide, dimethyl_a_	10,80	+	++	+	++	+		+	+					+		+	+
*Toluene_a_	11,72	+											+		+		+
*Butanoic acid, 2-methyl-methyl ester_a_	11,96																+
**S-methyl-propanethioate**_a_	12,98		+	+	+												
**Butanoic acid-3-methyl_a_	14,20						+					+	+		+		
**Butanoic acid-2-methyl_a_	14,71														+		
**Acetic acid, 3-methylbutyl ester**_a_	16,72													+	++		
**2-Heptanone**_a_	17,50					+	+	+	+								
**Butanethioic acid-S-methyl ester**_a_	17,55			+	+												
**Unknown-1**	17,94				+												
**Thiopivalic acid**_a_	20,34		+											+			
*Phenol_a_	22,05	+	+	+	+			+	+					+			
**Trisulfide, dimethyl**_a_	22,39		+	+	+			+	+					+			
**Unknown-2**	25,15							+									
**Benzeneacetaldehyde**_a_	26,03									+	+	+		+	+	+	
**1-Octanol**_a_	26,96			+	+												
**Hexanethioic acid, S-methyl ester**_a_	28,40				+												
*Phenylethyl alcohol_a_	29,67			+	+	+	+	+	+				+	+	+		
**Benzylmethyl cetone**_a_	30,47	+	+						+								
**Sulfide-1**	30,69				+												
**Benzeneethanol-a-methyl**_a_	30,72		+														
**Nonanone**_a_	31,55							+	+								
**Alcohol-1**	32,13				+												
**Tetrasulfide, dimethyl**_a_	35,51				+												
**Undecanone**_a_	36,51							+	+								
**1-Decanol**_a_	37,11	+		+	+			+									
*Indole_a_	38,79	+	+	+++	++	+	+	++	+	++	+	+	+	+++	+	+	+
**Cyclopentaneundecanoic acid, methyl ester**_a_	39,54									+							
**Aromatic-1**	41,48										+						
**1-Undecanol**_a_	41,75				+												
**Benzyl cetone unknown**	41,79	+	+														
**Alcohol-2**	45,68				+												
**Alcohol-3**	46,19			+	+												
**Alcohol-4**	46,73				+												
**Cetone-4**	47,19			+	+												
**Tetradecanone**_a_	54,30			+													
**Aromatic with nitrogen**	55,24			+	+												
*Hexadecanoic acid, methyl ester_a_	56,48				+				+								
**Methyl ester-1**	62,19				+				+								

^a^ = Compounds identified based on comparison of mass spectra with NIST14 Mass Spectral Library and analysis of their EI fragmentation partners.

* = VOCs reported as emanations from human skin by Bernier et al. (2000) [7].

** = VOCs reported as released by bacteria isolated from human skin by Verhulst et al. (2009) [8]. Ret time (min) = Retention time in minutes.

Average area percentage for each sample is represented as +++. High intensity: 50–100%, ++; medium intensity: 20–50%, +; low intensity: 0.01–20%. Be_e_ = *B*. *epidermidis* exponential phase, Be_s_ = *B*. *epidermidis* stationary phase, Ck_e_ = *C*. *koseri* 6P exponential phase, Ck_s_ = C. *koseri* 6P stationary phase, Dn_e_ = *D*. *nishinomiyaensis* 9C exponential phase, Dn_s_ = *D*. *nishinomiyaensis* 9C stationary phase, Ml_e_ = *M*. *luteus* 23 exponential phase, Ml_s_ = *M*. *luteus* 23 stationary phase, Sc_e_ = *S*. *capitis* 11C exponential phase, Sc_s_ = *S*. *capitis* 11C stationary phase, Sk_e_ = *S*. *caprae* 7P exponential phase, Sk_s_ = *S*. *caprae* 7P stationary phase, Se_e_ = *S*. *epidermidis* exponential phase, Se_s_ = *S*. *epidermidis* stationary phase, Sw_e_ = *S*. *warneri* exponential phase, Sw_s_ = *S*. *warneri* stationary phase.

**Fig 3:**
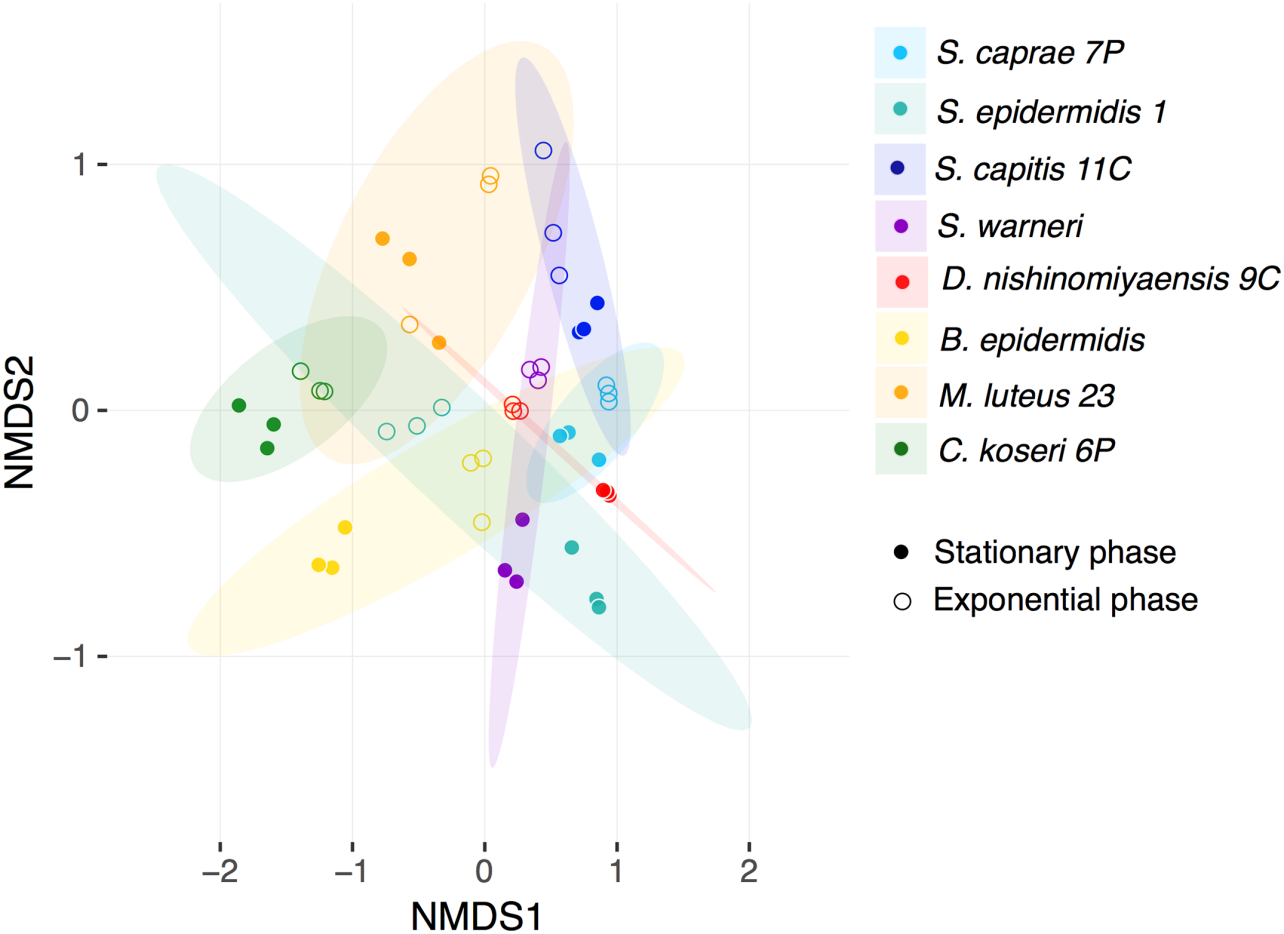
NMDS of VOC profiles from eight bacterial species isolated from human facial skin and cultured *in vitro* to two different growth phases. The analysis uses Bray-Curtis distances as a measure of dissimilarity. Stress value = 0.1776. Ellipses for each bacterial species (*S*. *caprae* 7P, *S*. *epidermidis* 1, *S*. *capitis* 11C, *S*. *warneri*, *D*. *nishinomiyaensis* 9C, *B*. *epidermidis*, *M*. *luteus* 23, *and C*. *koseri* 6P) describe a confidence interval of 95% using *t* distribution.

An NMDS plot was obtained for the VOCs only released by bacteria (Fig 3). To ensure the outcome we established the following two criteria: (1) the VOC was absent in the control analysis; and (2) the mass spectrum of the annotated compound corresponded to a bacterial VOC. NMDS was accurate in spatially representing the similarity/dissimilarity indices; the stress value obtained was 0.1776. To determine whether the bacterial species, the growth phase or both were the variables affecting the VOC profiles produced by skin bacteria, we considered all three variables in the NMDS analysis. Taking into consideration the bacterial community structure, the differential VOC profiles are better explained through the species-phase interaction (ANOSIM, R = 0.7635, p = 0.001). Furthermore, when considered separately as independent variables, i.e., species (ANOSIM, R = 0.5111, p = 0.001) and growth phase (ANOSIM, R = 0.05446, p = 0.035), both variables were also statistically supported as being factors involved in the differential VOC profiles. Despite the overlapping showed between ellipses representing the CI 95% using *t* distribution for each species (Fig 3); we found that this grouping variable explains at least 51% of the variance observed in the chemical profiles of the evaluated bacteria according to R^2^ estimations. Ellipses for the interaction species-phase and behavioral responses were not represented because at least four replicates (instead or three) per grouping variable are needed to perform this task. The VOC profiles of all *Staphylococcus* bacteria were found to be similar to each other with the exception of *S*. *epidermidis*, which, in the exponential phase, has a VOC profile more similar to bacteria of another genus. Although *B*. *epidermidis* belongs to a different genus of bacteria, it had a VOC profile similar to most of the *Staphylococcus* strains in the exponential phase.

**Fig 4:**
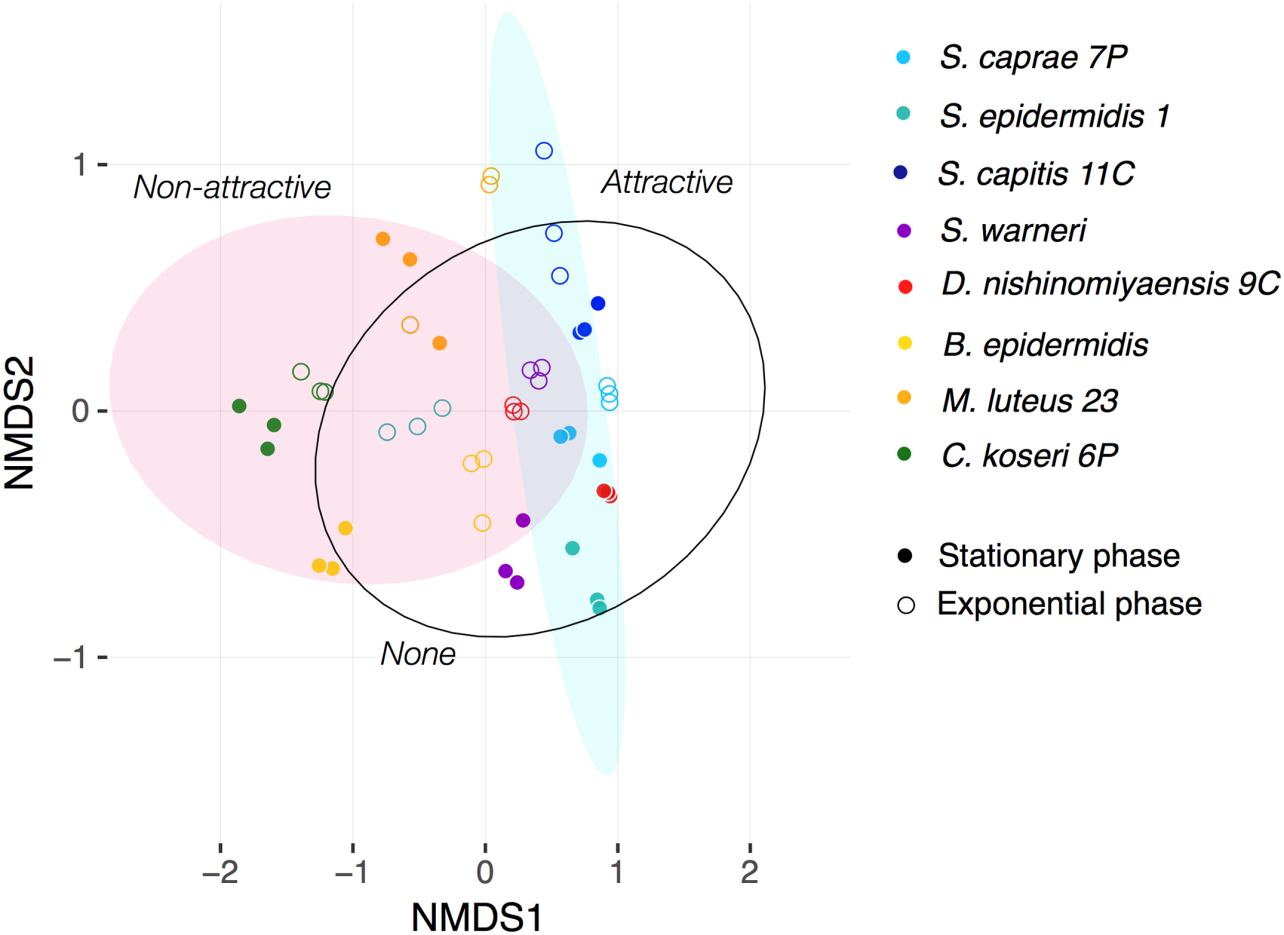
NMDS of VOC profiles from bacteria. Ellipses, according to the response of *R*. *prolixus* in behavioral experiments (Attractive/none/non-attractive), describe a confidence interval of 95% using *t* distribution.

Having collected evidence of a differentiation in the VOC profiles between bacterial species and growth phases, we went on to determine if this differentiation also correlates with the results of the behavioral experiments conducted with *R*. *prolixus*. Using the same NMDS but plotting ellipses for the “attractive”, “none” and “non-attractive” categories based on the results obtained in the behavioral experiments (Fig 4), we found a statistically supported differentiation (ANOSIM, R = 0.2407, p = 0.002) despite overlaps in the VOC profiles of bacteria in the “none” category with those of bacteria in the other categories. The ellipses are representing CI 95% using *t* distribution for each behavioral response, and they explain 24% of the variance observed in the chemical profiles of the tested bacteria according to R^2^ estimations. The larger overlapping in the ellipses is highlighting the differences in the explaining power of each grouping variable: bacterial species vs. behavioral response; and for that reason, they are not strictly assigning behavioral responses to the bacterial species (Fig 4).

## Discussion

We show here that bacteria isolated from human facial skin produce *in vitro* VOCs that could mediate the interaction between *Rhodnius prolixus* and humans depending on the VOCs released by individual bacterial species and its growth phase. These results support the tripartite interaction *R*. *prolixus*-bacteria-humans that we have shown previously in our group, as playing potentially a role in the vectorial transmission of Chagas disease [17, 18].

Molecular data have shown the presence of 205 identified genera of bacteria on human skin [24]. However, the diversity of bacteria revealed by culture-based methods is smaller compared to molecular methods [24]. We isolated 36 different morphotypes of bacteria from the skin of human volunteers, and seven species were identified (See S1 Table and S1 Fig). From the species isolated, we discarded the isolate *Propionibacterium acnes* from our behavioral experiments due to the difficulty in obtaining a growth curve for this bacterial species and the difficulty in using these bacteria in further behavioral experiments with *R*. *prolixus* under low oxygen conditions.

The other six bacteria isolated from the facial skin of our human volunteers are well-known as parts of the human skin microbiota [24, 25] with the exception of *Citrobacter koseri* (Proteobacteria: Enterobacteriaceae), which has been sporadically isolated from human skin and has also been reported as the cause of neonatal meningitis [26]. *S*. *epidermidis* was the only species isolated from all our volunteers; the identity and number of other species isolated for each volunteer exhibits high variation, as expected based on studies of skin microbiota among individuals [24, 25].

Skin bacteria isolated from human volunteers produce odors involved in the interaction between vectors of tropical diseases and humans [11]. Here, we reported for the first time the effect of VOCs released *in vitro* by bacteria isolated from human facial skin on the behavior of *Rhodnius prolixus*. In addition, our multivariate analysis confirm the differentiation in profiles of the VOCs released *in vitro* by bacterial species-growth phase (see Fig 3) as has been previously reported by Thorn *et al*. (2011) by using Selected Ion Flow Tube Mass Spectrometry (SIFT-MS) [27].

Our behavioral experiments showed *Rhodnius prolixus* to be attracted to VOCs released by *S*. *epidermidis* 1, *S*. *warneri* and *S*. *capitis* 11C (see Figs 2 and 4), while non-attractive behaviors were observed with *B*. *epidermidis*, *M*. *luteus* 23 and *C*. *koseri* 6P (see Figs 2 and 4). According with our eight isolated bacteria, most of the *Staphylococcus* species are attractive to *R*. *prolixus*, while the other genera of bacteria tested here are non-attractive except for *Dermacoccus nishinomiyaensis* 9C (see Fig 2). Our results confirm that 24% of the variation found in the profiles of the VOCs can be explained by the behavioral responses according to ANOSIM analysis. Even though, non-attractive ellipse representing CI of 95% overlap with *S*. *epidermidis 1*-exponential and *B*. *epidermidis*-stationary, and attractant ellipse CI overlap with *S*. *capitis 11C*-stationary, *D*. *nishinomiyaensis*-both phases and *S*. *caprae*-stationary, which are classified as without effect on the behavioral analysis (See Fig 4). Contrary to our results, behavioral experiments carried out with *An*. *gambiae* showed that higher attractiveness can be obtained to VOCs released by genera of bacteria other than *Staphylococcus* [11]; in fact, no bacteria showed any degree of non-attraction when tested with *An*. *gambiae* [11]. No statistically significant effect was observed on the behavior of *R*. *prolixus* to VOCs released in both growing phases by *D*. *nishinomiyaensis* 9C and *S*. *caprae* 7P (see Fig 2).

To date, behavioral experiments conducted on *Anopheles gambiae*, *Aedes aegypti* [9, 11] and *R*. *prolixus* (see Fig 2) highlight the role of the VOCs released by *S*. *epidermidis* in attracting blood-sucking insects. Additionally, it seems that VOCs released by the bacteria during the stationary phase of growth are affecting the attraction of *An*. *gambiae* [11] and *R*. *prolixus* (see Fig 2). From the other bacterial species isolated from human facial skin, only the VOCs released by *B*. *epidermidis* have been tested with *An*. *gambiae* [11] and *R*. *prolixus* (see Fig 2); the results obtained highlight the differences between these two blood-sucking insects. *B*. *epidermidis*, in stationary phase, was highly attractive to *An*. *gambiae* [11], while for *R*. *prolixus*, the exponential phase of the bacteria was non-attractive, and the stationary phase showed no behavioral response (see Fig 2). *Bacillus* spp. not tested in *R*. *prolixus*, have showed also *in vitro* attraction to *Anopheles* and *Aedes* mosquitoes [11, 28].

Among the VOCs released *in vitro* by the bacteria isolated, indole and CO_2_ were the only two compounds produced by all the species tested here (see S1 Table and S1 Fig). CO_2_ plays an important role in the attraction of blood-sucking insects [29], including Triatominae [17, 30]. However, Verhulst *et al*. (2009) [8] showed that the amount of CO_2_ released *in vitro* due to bacterial metabolism is not in the range required (1500–2300 ppm) to induce behavioral responses in *R*. *prolixus* [17]. According to the literature in *Anopheles*, indole is a volatile compound released by the microbiota in the oviposition sites, and indole is a major component of sweat and breath in humans [31, 32, 33]. The importance of this volatile compound is highlighted by the fact that detection of this compound by the antennae of *An*. *gambiae* is mediated by the odorant binding protein 1 (AgamOBP1) [34]. The role of indole in the behavior of *R*. *prolixus* is not known, and extrapolation of behavioral information obtained in experiments with mosquitoes should be considered with caution.

Bernier *et al*. (2000) identified 303 VOCs from human skin [7], from these, we observed that our bacteria released *in vitro* nine out of the 34 identified (see Table 1). In addition, Verhulst *et al*. (2009) identified 11 VOCs released *in vitro* by bacteria isolated from human skin [8]. In our bacteria we identified also three of these VOCs; however, none of them have been reported as VOCs released by human skin (see Table 1). Both results confirm that some of the VOCs released by our bacteria *in vitro* are normal constituents of the human skin emanations, and some others are product only of the *in vitro* metabolism.

The results shown in Fig 4 suggest that behavioral responses in *R*. *prolixus* are highly dependent on the complex mixture of VOCs released by bacteria and not on single compounds. Something similar was shown with behavioral experiments for *Anopheles* [8]. In the case of *R*. *prolixus* we found that benzeneacetaldehyde, which is the only compound different from CO_2_ and indole shared by the three attractive phase-bacteria (*S*. *epidermidis* 1-stationary, *S*. *warneri*-exponential and *S*. *capitis* 11C-exponential), can be found also in other *Staphylococcus* species but without behavioral responses (see Table 1). Something similar occurs also with dimethyl disulfide and phenol, which are shared by the four non-attractive bacteria-phase (*B*. *epidermidis*-exponential, *M*. *luteus 23*-stationary and *C*. *koseri 6P*-both phases), but they are also part of the chemical profile of other bacteria-phase which in the behavioral analysis showed none, or even attraction (see Table 1). In addition, the concentration at which the bacteria produce a VOC in a mixture should have an effect on the behavioral response of *R*. *prolixus*. These findings warrant further analysis of specific VOCs to analyze their ecological function in *R*. *prolixus*, because the behavioral response to bacteria seems to be very complex and seems to be dependent on the simultaneous interaction of several VOCs. Possible synergistic effects [35], acting agonistically or antagonistically, could explain these findings.

The results presented here support the hypothesis that VOCs produced by bacteria from the human facial skin microbiota differentially influence the behavioral response of *R*. *prolixus* [17]. These findings highlight the potential of bacterial VOCs for biotechnological use. Future behavioral experiments to test the attraction and/or repellency of *R*. *prolixus* to mixtures of VOCs are recommended; identified mixtures can be used as baits or repellents to avoid *Rhodnius prolixus*-humans contact and reduce the risk of vectorial transmission of Chagas disease mediated by *Rhodnius prolixus*.

## Supporting information

S1 Fig Bacterial strains isolated and identified from the facial skin of ten human volunteers from Bogota, Colombia. Squares marked with an “X” show the bacterial strains that were randomly selected to conduct the behavioral experiments with *Rhodnius prolixus*.
(EPS)

S2 Fig Growth curves of the bacteria evaluated. Red curves show the CFU/mL and blue curves show the absorbance at 620 nm.
(EPS)

S1 Table Bacterial morphotypes isolated from the facial skin of ten human volunteers from Bogota; identification with BLAST is also shown. (PDF)
