# Supplementary material for: Behavioral responses of Rhodnius prolixus to volatile organic compounds released in vitro by bacteria isolated from human facial skin
Source: PLoS Negl Trop Dis. 2018 Apr 23;12(4):e0006423. doi: 10.1371/journal.pntd.0006423 (PMC5933807; doi:10.1371/journal.pntd.0006423)
Supplement: S1 Table — (PDF) [file pntd.0006423.s003.pdf]

Volunteer	Morphotype	Identification	Accession number	Query coverage	E value	Maximum identity
1	9C	*Dermacoccus nishinomiyaensisp artial 16S rRNA gene, isolate PC IW01*	AM992178.1	95%	0.0	97%
	22C	*Staphylococcus epidermidis strain CIFRI CH-TSB-28 16S ribosomal RNA gene, partial sequence*	JF784042.1	98%	0.0	99%
	45C	No gene amplification				
2	4C	*Staphylococcus epidermidis strain CIFRI H-TSB-12-ZMA 16S ribosomal RNA gene, partial sequence*	JF799903.1	97%	0.0	99%
	5C	*Staphylococcus epidermidis strain F71055 16S ribosomal RNA gene, partial sequence*	HQ908690.1	97%	0.0	99%
	17C	No gene amplification				
	18C	*Staphylococcus epidermidis gene for 16S rRNA, partial sequence, isolate: T7-3M*	AB617572.1	97%	0.0	97%
	19C	*Staphylococcus epidermidis strain FUA2078 16S ribosomal RNA gene, partialsequence*	JN102557.1	97%	0.0	98%
3	12C	*Staphylococcus epidermidis gene for 16S rRNA, partial sequence, isolate: T7-3M*	AB617572.1	96%	0.0	97%
	13C	*Staphylococcus capitis subsp. capitis gene for 16S rRNA, partial*	AB626127.1	75%	0.0	98%

		sequence, strain: JCM 2420				
	14C	No gene amplification				
	47C	No gene amplification				
	48C	No gene amplification				
4	1C	*Staphylococcus epidermidis strain FUA2078 16S ribosomal RNA gene, partial sequence*	JN102557.1	91%	0.0	98%
	2C	*Bacterium N10 16S ribosomal RNA gene, partial sequence*	GQ389785.1	91%	0.0	97%
	3C	*Staphylococcus epidermidis gene for 16S rRNA, partial sequence, isolate: T7-3M*	AB617572.1	98%	0.0	97%
	36C	Poor template quality				
	39C	*Staphylococcus epidermidis strain CIFRI H-TSB-12-ZMA 16S ribosomal RNA gene, partial sequence*	JF799903.1	72%	0.0	98%
5	23	*Micrococcus luteus strain PCSB6 16S ribosomal RNA gene, partial sequence*	FR750272.1	99%	0.0	99%
	24	*Micrococcus luteus strain 5N-5 16S ribosomal RNA gene, partial sequence*	HQ663910.1	100%	0.0	99%
	25	*Citrobacter koseri ATCC BAA-895, complete genome*	CP000822.1	99%	0.0	99%
	27	*Uncultured bacterium clone ncd2780a11c1 16S ribosomal RNA*	JF241869.1	99%	0.0	100%

		gene, partial sequence				
	28	Poor template quality				
	29	*Staphylococcus epidermidis gene for 16S rRNA, partial sequence, isolate: T7-3M*	AB617572.1	100%	0.0	99%
	30	Poor template quality				
	23C	*Staphylococcus epidermidis ATCC 12228, complete genome*	AE015929.1	97%	0.0	99%
	34C	*Propionibacterium acnes 16S ribosomal RNA gene, partial sequence*	AF154832.1	98%	0.0	99%
6	1	*Staphylococcus epidermidis clone G08 16S ribosomal RNA gene, partial sequence*	GU003866.1	99%	0.0	99%
	3	*Staphylococcus epidermidis gene for 16S rRNA, partial sequence, isolate: T7-3M*	AB617572.1	100%	0.0	100%
	2P*	*Staphylococcus epidermidis*	KX901299.1	68%	0.0	99%
	4P*	*Staphylococcus sp.*	DQ659036.1	66%	0.0	99%
	5P*	*Staphylococcus epidermidis*	HG799951.1	61%	0.0	99%
	6P*	*Citrobacter koseri*	CP017665.1	85%	0.0	100%
	24C	*Staphylococcus epidermidis strain F71055 16S ribosomal RNA gene, partial sequence*	HQ908690.1	94%	0.0	98%
	29C	Poor template quality				
	30C	*Propionibacterium acnes 6609, complete genome*	CP002815.1	92%	0.0	97%

7	11	*Citrobacter koseri ATCC BAA-895, complete genome*	CP000822.1	99%	0.0	99%
	7P*	*Staphylococcus caprae*	KU922215.1	96%	0.0	99%
	25C	*Staphylococcus epidermidis strain 200 16S ribosomal RNA gene, partial sequence*	*EU730936.1*	99%	0.0	99%
	31C	*Staphylococcus epidermidis partial 16S rRNA gene, strain BGHMC5*	FR797804.1	62%	0.0	98%
	32C	*Propionibacterium acnes 6609, complete genome*	CP002815.1	96%	0.0	99%
8	20C	*Uncultured bacterium clone HSD-c-Cel9 16S ribosomal RNA gene, partial sequence*	JN698671.1	97%	0.0	98%
	21C	*Uncultured bacterium clone ncd594b12c1 16S ribosomal RNA gene, partial sequence*	HM284072.1	96%	0.0	98%
	37C	*Staphylococcus epidermidis strain FUA2078 16S ribosomal RNA gene, partial sequence*	JN102557.1	97%	0.0	98%
	43C	No gene amplification				
	44C	No gene amplification				
9	15C	*Staphylococcus epidermidis strain C490 16S ribosomal RNA gene, partial sequence*	JF803547.1	99%	0.0	98%
	16C	*Staphylococcus epidermidis strain F71055 16S ribosomal RNA*	HQ908690.1	97%	0.0	99%

		gene, partial sequence				
	41C	Poor template quality				
	42C	No gene amplification				
10	10C	*Staphylococcus caprae strain BB2_4A 16S ribosomal RNA gene, partial sequence*	JN644490.1	96%	0.0	99%
	11C	*Staphylococcus capitis strain BBN2R-02d 16S ribosomal RNA gene, partial sequence*	FJ357599.1	98%	0.0	97%
	26C	*Staphylococcus epidermidis strain FUA2078 16S ribosomal RNA gene, partial sequence*	JN102557.1	97%	0.0	98%
	27C	No gene amplification				
	28C	*Staphylococcus sp. DGM MH2a 16S ribosomal RNA gene, partial sequence*	JF923458.1	87%	0.0	97%

*Isolated by Paola Castillo in her undergraduate practice in 2010.
